# Machine learning-based clinical outcome prediction in surgery for acromegaly

**DOI:** 10.1007/s12020-021-02890-z

**Published:** 2021-10-12

**Authors:** Olivier Zanier, Matteo Zoli, Victor E. Staartjes, Federica Guaraldi, Sofia Asioli, Arianna Rustici, Valentino Marino Picciola, Ernesto Pasquini, Marco Faustini-Fustini, Zoran Erlic, Luca Regli, Diego Mazzatenta, Carlo Serra

**Affiliations:** 1 Machine Intelligence in Clinical Neuroscience (MICN) Laboratory, Department of Neurosurgery, Clinical Neuroscience Center, University Hospital Zurich, University of Zurich, Zurich, Switzerland; 2 IRCCS Istituto delle Scienze Neurologiche di Bologna, Programma Neurochirurgia Ipofisi-Pituitary Unit, Bologna, Italy; 3 Department of Biomedical and Neuromotor Sciences (DIBINEM), University of Bologna, Bologna, Italy; 4 Azienda USL di Bologna, Anatomic Pathology Unit, Bologna, Italy; 5 Department of Experimental, Diagnostic and Specialty Medicine (DIMES), University of Bologna, Bologna, Italy; 6 University of Bologna, School of Medicine and Surgery, Bologna, Italy; 7 Azienda USL di Bologna, Bellaria Hospital, ENT Unit, Bologna, Italy; 8 Department of Endocrinology, Diabetology and Clinical Nutrition, University Hospital Zurich (USZ) and University of Zurich (UZH), Zurich, Switzerland

**Keywords:** Pituitary, Predictive analytics, Outcome prediction, Machine learning, Acromegaly, Neurosurgery

## Abstract

**Purpose:**

Biochemical remission (BR), gross total resection (GTR), and intraoperative cerebrospinal fluid (CSF) leaks are important metrics in transsphenoidal surgery for acromegaly, and prediction of their likelihood using machine learning would be clinically advantageous. We aim to develop and externally validate clinical prediction models for outcomes after transsphenoidal surgery for acromegaly.

**Methods:**

Using data from two registries, we develop and externally validate machine learning models for GTR, BR, and CSF leaks after endoscopic transsphenoidal surgery in acromegalic patients. For the model development a registry from Bologna, Italy was used. External validation was then performed using data from Zurich, Switzerland. Gender, age, prior surgery, as well as Hardy and Knosp classification were used as input features. Discrimination and calibration metrics were assessed.

**Results:**

The derivation cohort consisted of 307 patients (43.3% male; mean [SD] age, 47.2 [12.7] years). GTR was achieved in 226 (73.6%) and BR in 245 (79.8%) patients. In the external validation cohort with 46 patients, 31 (75.6%) achieved GTR and 31 (77.5%) achieved BR. Area under the curve (AUC) at external validation was 0.75 (95% confidence interval: 0.59–0.88) for GTR, 0.63 (0.40–0.82) for BR, as well as 0.77 (0.62–0.91) for intraoperative CSF leaks. While prior surgery was the most important variable for prediction of GTR, age, and Hardy grading contributed most to the predictions of BR and CSF leaks, respectively.

**Conclusions:**

Gross total resection, biochemical remission, and CSF leaks remain hard to predict, but machine learning offers potential in helping to tailor surgical therapy. We demonstrate the feasibility of developing and externally validating clinical prediction models for these outcomes after surgery for acromegaly and lay the groundwork for development of a multicenter model with more robust generalization.

## Introduction

Acromegaly is a rare, progressive disease, caused by an oversecretion of growth hormone (GH) and elevated levels of insulin-like growth factor 1 (IGF-1) in the bloodstream [1]. A GH-secreting pituitary tumor is the cause of acromegaly in more than 95% of patients and surgical treatment remains the first-line therapy in most cases [2].

There are many variables that play into the likelihood of surgical success and endocrinological remission, such as age, Knosp grade, repeat surgeries, or even different somatostatin receptor subtypes [3–5]. The more factors that come into play, the harder it gets for clinicians to take them and their interactions into account. Based on these patient features, machine learning (ML) can be implemented to tailor treatment to a patient’s individual characteristics in the era of “personalized medicine” [6]. It has become evident that ML has strong potential for outcome prediction and sometimes even outperforms statistical modeling techniques [7, 8].

The ability to predict the likelihood of outcomes such as gross total resection (GTR) and biochemical remission (BR) as well as complications that are clinically relevant such as intraoperative cerebrospinal fluid (CSF) leaks from simple information available pre-operatively would be beneficial in risk-benefit patient counseling and shared decision-making. For some complications such as intraoperative CSF leaks, modifiable risk factors could even be adjusted based on personal risk, and precautions such as lumbar drainage could be taken in individuals with a high predicted risk of CSF leaks [9]. For this reason, we aim to develop and externally validate clinical prediction models for outcomes after transsphenoidal surgery for acromegaly.

## Methods

### Overview

A registry of patients who underwent endoscopic transsphenoidal surgery from the Department of Neurosurgery, IRCCS Institute of Neurological Sciences of Bologna was used to train prediction models for GTR, BR, and intraoperative CSF leaks. External validation was then carried out using patient data from the Department of Neurosurgery, University Hospital Zurich. This study was conducted conforming to the methods of transparent reporting of a multivariable prediction model for individual prognosis or diagnosis (TRIPOD) statement [10].

### Data sources

Prospective databases from two centers were used for model development. All patients who underwent surgery for acromegaly using the endoscopic transsphenoidal approach in Bologna from August 1998 to January 2020, as well as from July 2013 to May 2020 in Zurich were retrospectively evaluated. Operative procedures and preoperative assessments were conducted as described in previous publications [11, 12]. The inclusion criterion was for one or more of the three outcome measures (GTR, BR, and CSF leaks) to be available. Exclusion criteria were transcranial or combined procedures.

### Outcome measures

The ML models were developed to predict the subsequent binary endpoints: GTR, BR, and intraoperative CSF leaks. The primary outcome was GTR. The extent of resection was measured in a 3-month postoperative volumetric MRI and calculated as the percentage-wise reduction of tumor volume compared to baseline tumor volume on preoperative MRI. An extent of resection of 100% was defined as GTR. All measurements were performed by a board-certified neurosurgeon with extensive experience in pituitary surgery and imaging and were continually entered into the prospective registry. BR was strictly defined as normalization of hypersecretion into the normal reference range as defined by accepted international guidelines [13]. BR was defined as postoperative HGH level random or after oral glucose tolerance test <0.4 μg/l with normalization of age-adjusted IGF-1 levels at least 12 weeks after surgery and no clinical signs of GH activity. Cases with persistent slightly elevation of IGF-1 levels were considered in remission if HGH level after OGTT was adequately suppressed and no clinical signs of hormonal activity were present. The HGH and IGF-1 were measured using the chemiluminescence-Immunoassay LIAISON^®^ hGH and LIAISON^®^ IGF-I, respectively. The analyses were performed on the Liaison XL-Machine (DiaSorin, Saluggia, Italy).

Note that supplemental treatment modalities such as medical and radiation therapy were also taken into account when calculating BR.

### Input variables

Furthermore, we collected the following baseline variables: age, gender, prior surgery, Hardy classification (sellar and suprasellar) [14], Knosp classification [15], and tumor size. The Hardy and Knosp classifications both describe tumor morphology and correlate with resectability: While the Hardy classification focuses on intrasellar growth patterns and suprasellar extension, the Knosp classification assesses risk of cavernous sinus invasion by considering parasellar tumor extension relative to the internal carotid arteries [14, 15]. We defined macroadenoma as tumor size greater than 10 mm [16].

### Model development and validation

Continuous data are reported as mean value ± standard deviation (SD), while categorical data are given as absolute numbers (percentages). Models were trained on data from Bologna, and subsequently externally validated in Zurich. Both data sets were randomly reordered and then checked for approximately equal class distribution. There was no need for recursive feature elimination as only a limited number of variables were purposefully used.

A wide range of ML algorithms was applied including traditional and Bayesian generalized linear models (GLM), generalized additive models, random forests, stochastic gradient boosting machines (GBM) and a shallow neural network. They were tuned according to the area under the receiver operating characteristics curve (AUC) in fivefold repeated cross validation with ten iterations. A k-nearest neighbor algorithm was trained in parallel, allowing imputation of any missing data [17]. Binarization of predicted probabilities was carried out using a threshold based on the closest-to-(0,1)-criterion [18] on the derivation cohort. Discrimination was assessed using AUC, accuracy, sensitivity, specificity, positive predictive value (PPV), and negative predictive value (NPV). We also assessed calibration intercept and slope. Nonparametric 95% confidence intervals (CI) of the discrimination and calibration metrics were computed in 1000 bootstrap resamples. Variable importance was assessed for each model using a universal AUC-based method, and importance measures were scaled from 0 to 100 for each model [19]. All evaluations were executed using R version 4.0.2 [20].

## Results

### Patient cohort

In total, 307 patients were used in the training process. The training data had no missing values apart from the age of a single patient. Mean age was 47.2 ± 12.7 years and 133 (43.3%) patients were male. GTR and BR were achieved in 226 (73.6%) and 245 (79.8%) patients, respectively, and CSF leaks occurred intraoperative in 38 (12.5%) of patients. The external validation cohort consisted of 46 patients, among whom there were 5 (10.9%) patients with incomplete data on GTR and 6 (13.0%) patients with incomplete data on BR. Only four (8.7%) patients in the external validation set had incomplete baseline data. Mean age was 47.5 ± 14.4 years and 22 (47.8%) patients were male. In the external validation cohort, GTR occurred in 31 (75.6%) patients, while BR occurred in 31 (77.5%). Intraoperative CSF leaks occurred in 12 (26.1%) patients in the external validation cohort. Detailed patient characteristics for both cohorts are provided in Table 1.

**Table 1:** Patient characteristics and incidence of outcomes

Variable	Cohort	
	Development (*n* = 307)	External validation (*n* = 46)
Male gender, *n* (%)	133 (43.3%)	22 (47.8%)
*No. missing*	*0 (0.0%)*	*0 (0.0%)*
Age [yrs.]		
Mean ± SD	47.2 ± 12.7	47.5 ± 14.4
Median (IQR)	55 (38–57)	46 (37–60)
Range	13–78	21–73
*No. missing*	*1 (0.3%)*	*0 (0.0%)*
Prior surgery, *n* (%)	49 (16%)	10 (21.7%)
*No. missing*	*0 (0.0%)*	*0 (0.0%)*
Hardy sellar, *n* (%)	236 (76.9%)	42 (91.3%)
Grade 1	514 (21.1%)	14 (30.4%)
Grade 2	324 (13.3%)	10 (21.7%)
Grade 3	243 (10.0%)	3 (6.5%)
Grade 4	121 (5.0%)	15 (32.6%)
*No. missing*	*0 (0.0%)*	*3 (6.5%)*
Hardy suprasellar, *n* (%)	174 (56.7%)	21 (45.6%)
Grade A	109 (35.5%)	13 (28.3%)
Grade B	20 (6.5%)	6 (13.0%)
Grade C	2 (0.7%)	1 (2.2%)
Grade D	3 (1%)	0 (0%)
Grade E	40 (13%)	1 (2.2%)
*No. missing*	*0 (0.0%)*	*1 (2.2%)*
Knosp classification, *n* (%)	96 (31.3%)	31 (67.4%)
Grade 1	24 (7.8%)	7 (15.2%)
Grade 2	27 (8.8%)	6 (13.0%)
Grade 3	30 (9.8%)	15 (32.6%)
Grade 4	15 (4.9%)	3 (6.5%)
*No. missing*	*0* (*0.0%*)	*0* (*0.0%*)
Macroadenoma, *n* (%)	199 (64.8%)	36 (80.0%)
*No. missing*	*0 (0.0%)*	*1 (2.2%)*
Gross total resection (GTR), *n* (%)	226 (73.6%)	31 (75.6%)
*No. missing*	*0 (0.0%)*	*5 (10.9%)*
Intraop. CSF leak, *n* (%)	38 (12.5%)	12 (26.1%)
*No. missing*	*0 (0.0%)*	*0 (0.0%)*
Biochemical remission, *n* (%)	245 (79.8%)	31 (77.5%)
* No. missing*	*0 (0.0%)*	*6 (13.0%)*

*SD* standard deviation, *IQR* interquartile range

### Model performance

#### Gross total resection

A detailed overview of model performance is provided in Table 2, including calibration metrics and training performance. At external validation, the GTR model (traditional GLM) achieved an AUC of 0.75 (0.59–0.88), 0.52 (0.33–0.70) for sensitivity and 0.90 (0.69–1.00) for specificity. The resulting PPV was 0.94 (0.82–1.00).

**Table 2:** Discrimination and calibration metrics of the machine learning-based prediction models

Outcome	Gross total resection		Biochemical remission		CSF leak	
Type of model	GLM		GBM		Bayesian GLM	
Metric	Development (*n* = 307)	External validation (*n* = 41)	Development (*n* = 307)	External validation (*n* = 46)	Development (*n* = 307)	External validation (*n* = 40)
Discrimination						
AUC	0.68 (0.66–0.70)	0.75 (0.59–0.88)	0.62 (0.59–0.64)	0.63 (0.40–0.82)	0.69 (0.67–0.72)	0.77 (0.62–0.91)
Accuracy	0.65 (0.63–0.67)	0.61 (0.46–0.75)	0.63 (0.61–0.64)	0.58 (0.42–0.72)	0.60 (0.58–0.62)	0.70 (0.57–0.83)
Sensitivity	0.65 (0.63–0.67)	0.52 (0.33–0.70)	0.64 (0.63–0.67)	0.61 (0.44–0.77)	0.71 (0.66–0.75)	0.58 (0.29–0.87)
Specificity	0.65 (0.61–0.68)	0.90 (0.69–1.00)	0.57 (0.53–0.61)	0.44 (0.12–0.80)	0.59 (0.57- 0.61)	0.74 (0.57- 0.88)
PPV	0.84 (0.82–0.85)	0.94 (0.82–1.00)	0.85 (0.84–0.87)	0.79 (0.61–0.95)	0.19 (0.17–0.22)	0.44 (0.20–0.69)
NPV	0.40 (0.37–0.43)	0.38 (0.18–0.57)	0.27 (0.26–0.31)	0.25 (0.06–0.47)	0.93 (0.92–0.95)	0.83 (0.69–0.96)
Calibration						
Intercept	0.97	1.49	1.29	1.14	−1.77	−0.64
Slope	0.52	0.03	0.58	0.76	0.39	0.68
Threshold	0.55		0.52		0.41	

Metrics are provided with bootstrapped 95% confidence intervals

*AUC* area under the curve, *PPV* positive predictive value, *NPV* negative predictive value

#### Biochemical remission

Our GBM achieved an AUC of 0.63 (0.40–0.82) on the external validation data, as well as a sensitivity of 0.61 (0.44–0.77) and specificity of 0.44 (0.12–0.80). A PPV of 0.79 (0.61–0.95) was reached.

#### Cerebrospinal fluid leaks

The Bayesian GLM used to predict CSF leaks displayed an AUC of 0.77 (0.62–0.91) at external validation, while a sensitivity of 0.58 (0.29–0.87) and a specificity of 0.74 (0.57–0.88) were recorded. The NPV reached 0.83 (0.69–0.96).

### Variable importance

**Fig. 1:**
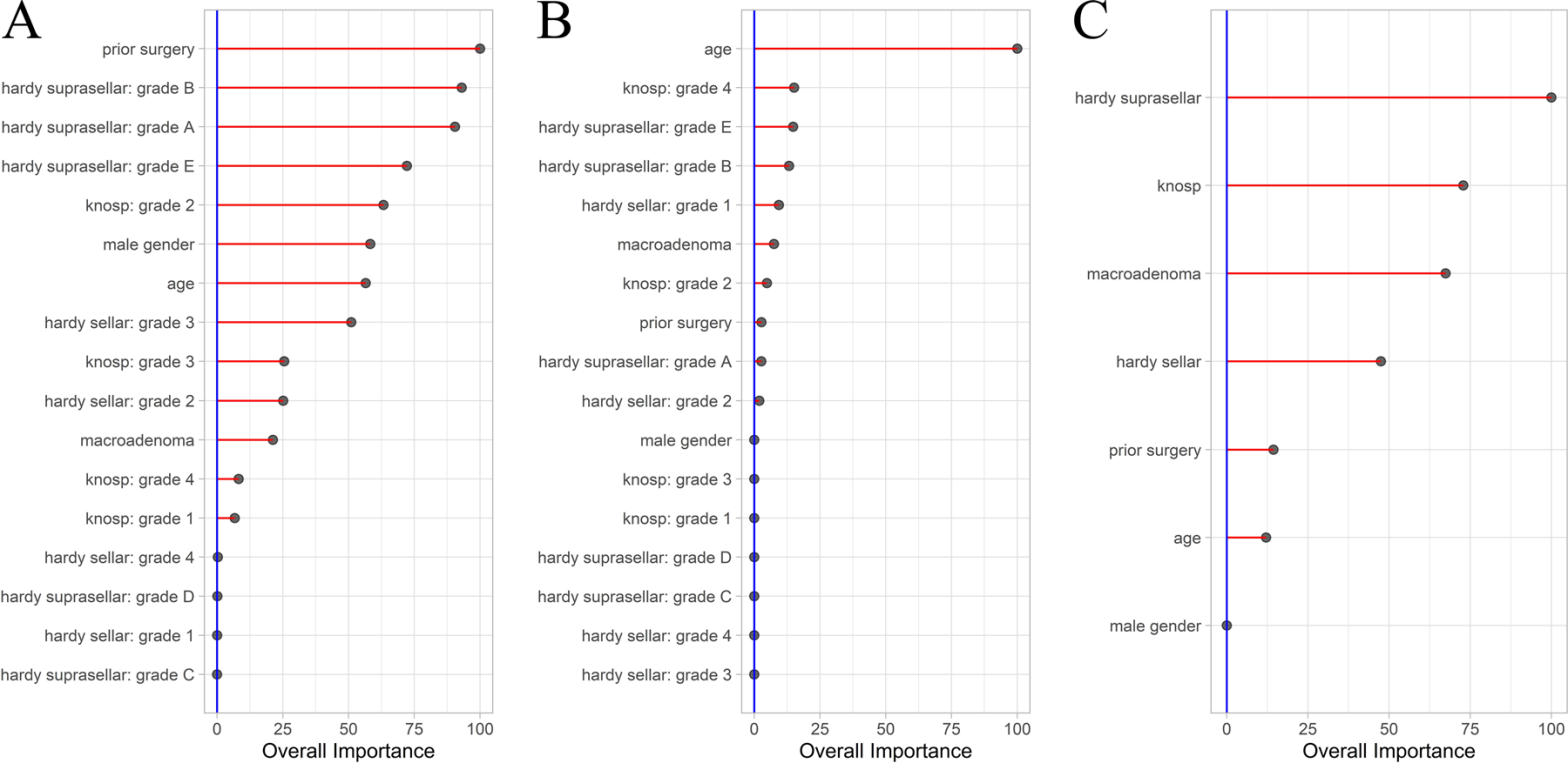
AUC-based variable importance for the three models. Importance values have been scaled from 0 to 100. **A** Gross total resection; **B** Biochemical remission; **C** Intraoperative cerebrospinal fluid leakage

Table 3 provides an overview of variable importance measures for each of the three models. For prediction of GTR, prior surgery and Hardy grading contributed most to predictions. Patient age and Hardy grading contributed most to predictions of BR. Last, Hardy and Knosp grading contributed most to predictions of intraoperative CSF leaks (Fig. 1).

**Table 3:** AUC-based relative variable importance in the machine learning-based prediction models

Variable	Gross total resection	Biochemical remission	CSF leaks
Male gender	58.24	0.00*	0.00*
Age	56.51	100.00	12.11
Prior surgery	100.00	2.71	14.37
Hardy sellar			47.47
Grade 1	0.02	9.36	
Grade 2	25.16	1.93	
Grade 3	51.03	0.00*	
Grade 4	0.30	0.00*	
Hardy suprasellar			100.00
Grade A	90.49	2.67	
Grade B	93.01	13.21	
Grade C	0.00*	0.00*	
Grade D	0.12	0.00*	
Grade E	72.18	14.77	
Knosp classification			72.88
Grade 1	6.71	0.00*	
Grade 2	63.30	4.81	
Grade 3	25.54	0.00	
Grade 4	8.19	15.14	
Macroadenoma	21.24	7.48	67.41

*Corresponds to a variable importance of 0.00, i.e., the variable was not included in the final model

## Discussion

In this study, the feasibility of predicting surgical and endocrinological outcome after transsphenoidal surgical treatment of acromegaly was evaluated. With data from two registries, three clinical prediction models were trained and subsequently externally validated. The achieved results proved to be promising and thereby displayed that there is significant potential for clinical application of ML.

In surgical treatment of acromegaly, normalization of GH levels through total resection is crucial. Treatment-refractory acromegaly puts patients at risk for early mortality [21]. Consequently, a more aggressive surgical approach is justified in refractory cases. It has been proven that the percentage of reduction in GH closely correlates with the fraction of removed tumor in surgery for acromegaly [22]. Further, low serum GH levels indicate persisting remission, whereas with higher levels the probability of recurrent disease—linked to significant mortality —is markedly larger [23, 24]. Even intraoperative CSF leaks are detrimental to endocrinological outcomes, since they have been shown to inhibit hormonal recovery after surgery—apart from their inherent risk for persisting CSF fistulas and meningitis [25, 26].

Surgical outcome depends on many variables that are hard to account for—including surgical experience, skill, and caseload [2]—making their prediction difficult. ML methods can deduce a simple risk assessment model from relatively complex data [4, 5]. For this reason, ML has been proven to aid in improved shared decision-making as well as enhanced patient care by modification of risk factors [7, 27, 28]. However, some factors cannot be taken into account by any model—prediction models will always remain just that: models of reality. Therefore, ML should never replace the careful study of imaging results, the contemporary literature, and surgical experience. Rather, it should be seen as supplemental information available to surgeons, complementing the existing evidence and allowing personalized risk-benefit assessment. There is decent evidence that ML can help with improved surgical decision-making, and in some cases may even outperform expert predictions [28].

Other important parameters that help physicians include simple scores and classifications, like the Knosp classification [29] or the Zurich Pituitary Score [30]. While these scores are well validated and robustly predict e.g. GTR, they are rather difficult to tailor to specific patient characteristics because they stratify patients into large risk groups. For the ML models established in this investigation, some of these classifications were combined with other recognized prognostic factors to deliver predictions that are precisely tailored to each patient. When trying to compare the performance of ML models with these scoring systems, little valid comparisons can be made, since reporting of performance measures such as sensitivity and specificity for these scores is uncommon. A systematic review by Dhandapani et al. [31] allows comparison to the raw Knosp classification and its relationship with GTR. This review found that the usual dichotomization of the Knosp classification (Knosp 1 and 2 vs. Knosp 3 and 4) led to a sensitivity of 66.4% and specificity of 90.3% for GTR [31]. Furthermore, in future studies, by combining additional endocrinological parameters like preoperative IGF-1 or early postoperative GH value in the model, a better performance for BR prediction might be obtained [32–34]. However, the rationale of this study was to develop a simple tool that can give meaningful predictions using basic, pre-operatively available data only.

The developed models demonstrated good generalizability, performing similarly well on the external validation data as compared to on the training data. The GTR and BR models had a high PPV, making them suitable as “rule-in” models. Conversely, the CSF leak model demonstrated a high NPV, and is thus more suitable in a “rule-out” setting.

A major criticism of ML-based prediction models is that they at times work like a “black box” [35]. Especially with deep neural networks, one is often confronted with the inability to understand why certain predictions have been made. By feeding the algorithm with the required data it can often provide precise outcome prediction, but it remains unknown how the internal decision-making process works. In this study, an initial problem was solved firstly by relying on algorithms with a complexity suitable to tabulated medical data. In addition, insight into the decision-making procedure can be gained by evaluating the variable importance listed in Table 3. With ML, interpretability can involve an inherent trade-off for better prediction power.

In conclusion, it can be stated that prediction of these complex outcomes like BR and GTR–which are certainly governed also by “unmeasurable” factors such as surgeon experience—from simple input data remains a difficult task, although ML can provide relatively accurate predictions in this pilot study already. Using more complex variables as input instead would probably improve the performance, but too complex inputs could be undesirable, as they would make the application of the models impractical. This study aimed at creating a simple tool that can give meaningful predictions using basic, pre-operatively available data. The models developed are proof that this is no longer mere wishful thinking. To the best of the authors’ knowledge, there are no other published, externally validated clinical prediction models for outcomes of transsphenoidal pituitary surgery in acromegalic patients. Once these models are enhanced by additional patient data and more participating centers to foster generalizability, an integration into a web application available to the public would be feasible.

### Limitations

The main limitation of our study is the relatively low sample size. Although a very decent surgical cohort of over 300 acromegalic patients was included for training— one of the largest contemporary single-center cohorts in the literature— this sample size is still rather low for ML. For example, evaluation of model calibration usually requires larger amounts of data. Recalibration would not change anything in this respect, and would only artificially improve calibration [36, 37]. Larger amounts of data would also likely improve general model performance. Even though external validation was carried out, which demonstrated generalizability of our models, including more participating centers to create a multicenter model that may account for the differences in surgical strategies, and so forth. Another important factor to consider is that these models are not applicable to centers with radically different treatment protocols. Importantly, surgical outcomes are also influenced by surgical experience and caseload [38], inherently limiting the generalizability of any prediction model, score, or classification for surgical outcome. For example, a significantly different endpoint incidence may lead to systematic over- or underestimation of the outcome probability from the developed models [36]. Furthermore, it needs to be taken into account that all clinical prediction models are unable to reliably predict extreme cases that fall outside the range of the training data (extrapolation) [39, 40]. Furthermore, our models are trained on “real-world” registry data. The rate of BR was higher than the rate of GTR due to supplemental treatments such as radiation and medical therapy. While this does represent the “real-world” clinical practice—with some patients undergoing multiple treatments—our models may be less suitable when aiming to predict the chances of BR purely from tumor resection. Problems may also occur because of the poor reliability between different physicians’ ratings [41, 42]. Especially with the Knosp and Hardy classification, there is evidence for poor inter-rater reliability.

## Conclusions

GTR, BR, and CSF leaks remain hard to predict, but ML may offer remarkable potential in helping to tailor surgical therapy. We demonstrate the feasibility of developing and externally validating clinical prediction models for these outcomes after surgery for acromegaly. This study lays the groundwork for development of a multicenter model with more robust generalization.

## Data Availability

The data in support of our findings can be obtained upon reasonable request from the corresponding author.

## Code availability

For the source code used to compute the presented results refer to the Supplement.
